# Non-Conventional Prognostic Markers in Life-Threatening COVID-19 Cases—When Less Is More

**DOI:** 10.3390/jcm13185369

**Published:** 2024-09-11

**Authors:** Martin Rozanovic, Kata Várady-Szabó, Kamilla Domokos, Tamás Kiss, Csaba Loibl, Gergely Márovics, Szilárd Rendeki, Csaba Csontos

**Affiliations:** 1 Department of Anaesthesiology and Intensive Care, University of Pécs Medical School, 7624 Pécs, Hungary; rozanovic.martin@pte.hu (M.R.); domokos.kamilla@pte.hu (K.D.); kiss.tamas@pte.hu (T.K.); csontos.csaba@pte.hu (C.C.); 2 Department of Public Health Medicine, University of Pécs Medical School, 7624 Pécs, Hungary

**Keywords:** inflammation, neutrophil/lymphocyte ratio, infection, hemorheology, COVID-19

## Abstract

**Background/Objectives**: In this study, we aimed to compare the predictive power of non-conventional (neutrophil/lymphocyte ratio—NLR; platelet/lymphocyte ratio—PLR) and conventional markers (C-reactive protein—CRP; procalcitonin—PCT; interleukin-6—IL-6) in terms of disease progression and mortality in severe SARS-CoV-2 patients. **Methods**: In this prospective observatory study, blood samples were collected daily, focusing on the established inflammatory markers. Critically ill COVID-19 patients who required ICU admission were included. Patient treatment followed established COVID-19 protocols, and the data analysis was performed using SPSS with non-normal distribution methods. The study cohort primarily included patients infected with the delta variant. **Results**: A mortality rate of 76.6% was observed among 167 patients during the study period. Significant differences in conventional and non-conventional markers between survivor and non-survivor groups were observed. The PCT levels were significantly elevated (*p* < 0.005) in the deceased group. Among the non-conventional markers, the NLR was consistently higher in non-survivors and emerged as a significant predictor of mortality, whereas the PLR was not elevated among the non-survivors. ROC analyses indicated that PCT and the NLR were the markers with the highest predictive power for mortality. The multivariate logistic regression analysis identified NLR, PCT, CRP, and IL-6 as significant predictors of mortality across different days. The NLR showed a consistent, though not always statistically significant, association with increased mortality risk, particularly on Days 2 and 5. **Conclusions**: The NLR’s accessibility and simplicity of determination make it a valuable and practical tool for monitoring inflammatory processes in viral infections. Our findings suggest that incorporating NLR analysis into routine clinical practice could enhance the early identification of high-risk patients, thereby improving patient management and outcomes.

## 1. Introduction

Prognostic blood markers for sepsis and severe sepsis are in high demand, as a significant number of COVID-19 patients have progressed to severe pneumonia, pulmonary oedema, acute respiratory distress syndrome, or multiple-organ failure, ultimately resulting in high death rates [1,2].

Severe cases of COVID-19 manifesting as ARDS are characterised by dyspnoea and severely reduced blood oxygen levels [2]. Patients may develop additional bacterial and/or fungal infections, which could evolve into severe septic conditions [3]. ARDS is a primary cause of respiratory failure and is responsible for 70% of COVID-19-related deaths [2]. Quantitative CT COVID scores, combined with laboratory markers, help clinicians make informed decisions about the rapid progression of COVID-19. Follow-up monitoring and lab results provide insights into changes in the condition of COVID-19 patients [4].

One of the key factors contributing to the severity of COVID-19 is the inflammatory response triggered by the virus in infected individuals [5]. COVID-19 primarily affects the respiratory system and can range from mild symptoms to severe respiratory distress and multi-organ failure. Upon infection, SARS-CoV-2 replicates within host cells, leading to the release of viral particles and activation of the immune response [5].

The immune response plays a crucial role in combating viral infections. However, in some COVID-19 patients, an excessive and dysregulated inflammatory response occurs, leading to a phenomenon known as a “cytokine storm”, which is the immune system’s excessive release of cytokines in response to a viral infection. Cytokines are small signalling proteins that regulate immune responses, but the overproduction of pro-inflammatory cytokines can lead to tissue damage and organ dysfunction [6]. Several factors contribute to dysregulation of the immune response to COVID-19. SARS-CoV-2 infection induces the release of various cytokines, depending on the patient’s symptom severity. If the patient presents with mild or moderate symptoms, then IL-6, IL-10, and TNF-α are released in higher numbers. On the other hand, when severe symptoms occur, in addition to these cytokines, IL-2 and MCP-1 are also secreted in an uncontrolled manner [7,8]. These cytokines attract immune cells, such as macrophages and T cells, to the site of infection. However, an excessive influx of immune cells can lead to collateral tissue damage [5,6,9]. Therefore, a dysregulated inflammatory response is associated with severe disease manifestations and poor outcomes. Recent research supports the potential of studying classical inflammation-related markers, particularly focusing on detailed analysis of cytokine profiles and their dynamics. It has been shown that certain cytokines, such as IL-6, IL-8, and G-CSF, exhibit significantly higher levels in severe COVID-19 cases, especially in patients requiring invasive mechanical ventilation. These findings suggest that cytokine patterns could serve as prognostic markers, helping to tailor glucocorticoid therapy more effectively and potentially improving patient outcomes by preventing progression to more severe respiratory failure [10,11].

Potential secondary infections could trigger a cytokine storm, contributing to sepsis symptoms, which result in death in 25–30% of COVID-19 cases [3,12,13]. In these instances, dysregulated inflammation causes damage to multiple organs, particularly the heart, liver, and kidneys, culminating in multiple-organ failure. These complications include acute kidney injury, cardiac dysfunction, and clotting disorders. It is worth noting that many individuals with SARS-CoV-2 infection who progressed to renal failure ultimately met a fatal outcome [9]. A decrease in the lymphocyte count is frequently seen in COVID-19 patients, especially those with severe disease. Lymphocytes play a crucial role in the immune response, and their reduction suggests impaired immune function. Neutrophilia, an increase in the number of neutrophils, is commonly observed in severe cases of COVID-19 and can be a marker of systemic inflammation [14].

Recent research supports the potential of studying classical cell-related, inflammation-related indicators, such as quantifying white blood cell and thrombocyte counts, as indicators of blood quality. The neutrophil/lymphocyte ratio (NLR) and the platelet/lymphocyte ratio (PLR) are gaining new focus as valuable tools for diagnosing and predicting the outcome of different infectious illnesses, including COVID-19 infection [14,15,16,17,18]. Utilising simple and common variables, such as neutrophil and lymphocyte counts, as reliable prognostic markers could significantly improve personalised therapies and treatment settings. For instance, the authors of a recent study emphasised that the early discharge of patients with severe disease is feasible if patient selection is optimal [19,20].

In our study, we compared the predictive value of these non-conventional (NLR and PLR) and conventional markers (CRP, PCT, and IL-6) regarding disease progression and mortality in SARS-CoV-2-infected patients. Additionally, we aimed to explore the alterations in these markers in other virus-caused infections, underlining the novelty of utilising the NLR and PLR as significant predictors in the context of severe viral infections such as COVID-19.

## 2. Materials and Methods

In this prospective study, 167 patients were enrolled from February 2021 to March 2022 at University of Pécs. All consecutive patients during the study period underwent daily blood sample collection, where the focus of the blood analyses was on the established inflammatory markers CRP, PCT, and IL-6. Samples were also taken for conventional qualitative checks of white blood cells and thrombocytes. Ratios based on the raw cellular data, such as the NLR and PLR, were also examined. Established COVID-19 protocols were followed in patient treatment. SPSS was utilised for the data analysis with non-normal distribution methods.

### 2.1. Inclusion/Exclusion Criteria

Adult individuals admitted to the intensive care unit were enrolled in this study based on strictly defined criteria: PaO_2_/FiO_2_ < 200, mean arterial pressure (MAP) < 60 mmHg, a need for norepinephrine, and a requirement for mechanical ventilation due to SARS-CoV-2 infection confirmed with PCR testing. Additionally, a CT scan performed on these patients revealed pneumonia, as described by a radiologist. The exclusion criteria were as follows: patients with severe heart failure classified as NYHA IV, individuals with liver cirrhosis or portal hypertension, those with prolonged reliance on haemodialysis, those with blood-related malignancies, and those undergoing long-term immunosuppressive treatment. Furthermore, patients in whom pneumonia was not confirmed with a CT scan were excluded from this study. None of the patients had received vaccination against SARS-CoV-2 infection.

### 2.2. Location and Design

The first blood sample was taken upon admission to the intensive care unit (Day 1). Subsequent samples were gathered each day between 5 AM and 6 AM throughout the 5-day study period in the ICU.

### 2.3. Laboratory Analysis

CRP, PCT, and IL-6 were analysed in the Department of Laboratory Medicine, University of Pécs, similar to the total blood count and qualitative blood indicators. The NLR was determined by dividing the number of neutrophil granulocytes by the number of lymphocytes. The PLR was determined by dividing the number of platelets by the number of lymphocytes. All cell counts were obtained from routine daily lab reports.

### 2.4. Patient Demographics

A total of 167 ICU patients with SARS-CoV-2 infection were included in this study. The median age was 69 years (46–85). The median age of the 39 surviving patients (SU) was 65 years (30–80). For the 128 patients who died in the ICU (NS), the median age was 71 years (47.35–85.55). Mortality was 76.6% in this population, but it did not correlate with overall COVID-19 mortality in our clinic. The daily breakdown of deaths was 60, 7, 7, and 54. The sample was composed of 104 male and 63 female patients.

### 2.5. Treatment

All patients received their treatment based on our department’s established COVID-19 therapy protocol (Department of Anaesthesiology and Intensive Therapy, Clinical Centre, University of Pécs). The key components of this protocol were as follows:

- All patients were subcutaneously given a preventive dose of low-molecular-weight heparin (LMWH) in enoxaparin.
- Dexamethasone (12 mg) was administered intravenously to all patients upon admission to the intensive care unit.
- Preventive antibiotic treatment was not employed.
- Each patient received remdesivir intravenously, with a loading dose of 200 mg on Day 1, followed by 100 mg for the subsequent four days.
- Biological therapy was not utilised in any of the patients.
- The preventative administration of platelet aggregation inhibitors was not part of the treatment strategy.
- Organ support therapy was performed as required (IRRT, CRRT, mechanical ventilation, and so on).

### 2.6. Ethics Approval and Consent

This prospective observatory study was conducted in a 27-bed intensive care unit (ICU) from February 2021 to March 2022 at University of Pécs, Hungary. All patients received treatment in line with both Hungarian and global medical directives. For all consecutive patients, for inclusion in this study, we obtained written consent from either the participants themselves or their legal representatives. The patients’ data were handled in accordance with GDPR data protection. Ethical clearance was obtained from the University of Pécs’ ethics committee (protocol number: 8581–PTE 2021).

### 2.7. Statistical Analysis

The compiled data were analysed using the Statistical Package for the Social Sciences (SPSS) version 28.0, developed by IBM Corporation, Armonk, NY, USA. The presented values are expressed as the medians and interquartile ranges (IQRs) due to the absence of a normal distribution. Intergroup variations were examined using the Mann–Whitney U-test. Notable patterns were identified by applying the Wilcoxon test across the study days. A multivariate logistic regression analysis was performed to study which factors were independent predictors of mortality. An ROC analysis was performed to confirm the results of the multivariate logistic regression. The level of statistical significance was set to *p* < 0.05 for all tests.

## 3. Results

Survival was the principal selector in our analysis of 167 patients. A subgroup analysis of 39 survivors (discharged from the ICU alive) and 128 deceased patients was performed. The need for renal replacement therapy developed in 29 patients, necessitating some form of renal replacement therapy. Based on the medical histories of the included patients, we categorised them according to their known comorbidities (Table 1).

### 3.1. Data Analysis

#### 3.1.1. Comparison of ICU Survivor and Non-Survivor Groups

**Figure 1:**
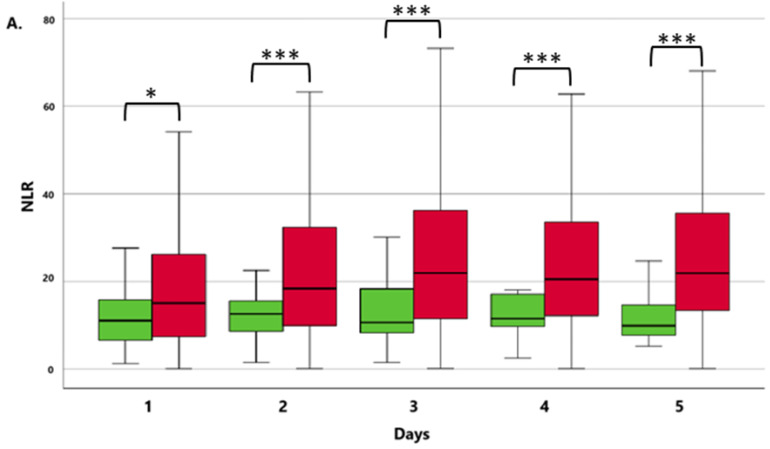

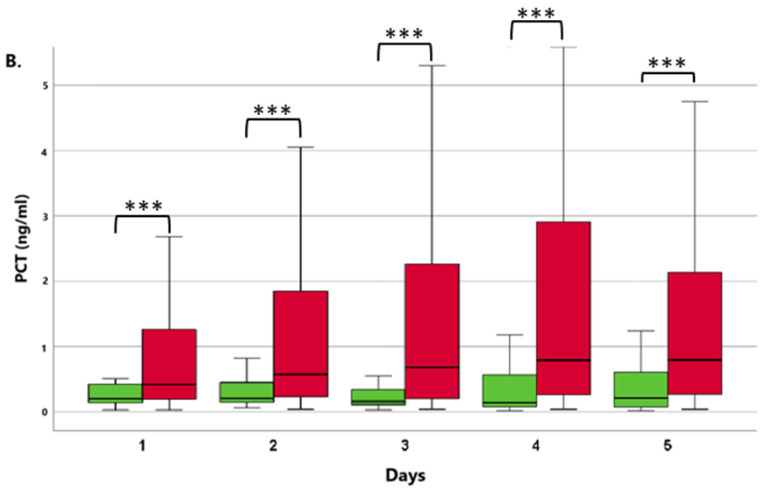
The kinetics of the median NLR (**A**) and PCT (**B**) levels between survivor and non-survivor patients. Green columns: surviving patients; red columns: non-surviving patients. * *p* < 0.05 and *** *p* < 0.005 between the NSU and SU groups. Data are presented as medians in 25–75% interquartile ranges and 5–95% confidence intervals.

Among the conventional parameters studied, PCT (Figure 1B) was significantly higher in the non-survivor group on all days of the examination (*p* < 0.005). As for IL-6, significantly elevated values were observed on Days 4 and 5 (*p* < 0.005). Concerning the non-conventional markers examined, the NLR consistently showed significantly higher values in the non-survivor patient group each day (Figure 1A), whereas for the PLR, no significant differences were observed between the two groups. A comparison of the examined markers in the non-surviving group revealed that the widely used PCT indicator (Figure 1B), employed in everyday clinical practice, increased from the first to the fourth day, with the highest values recorded on the fourth day. This trend closely paralleled that of the NLR (Figure 1B), which increased in the first three days and peaked on the third day. In contrast, CRP did not exhibit an increasing trend, with the highest value recorded on the first day. When comparing markers between the groups of survivors, it was observed that CRP levels were also highest on the first day and then gradually decreased. At the same time, PCT increased during the first two days, reaching its peak value on the second day in the survivor group. This pattern closely aligned with that of the NLR, which increased during the first two days, reaching its highest value on the second day.

#### 3.1.2. Patients with or without the Need for Renal Replacement Therapy (RRT)

**Figure 2:**
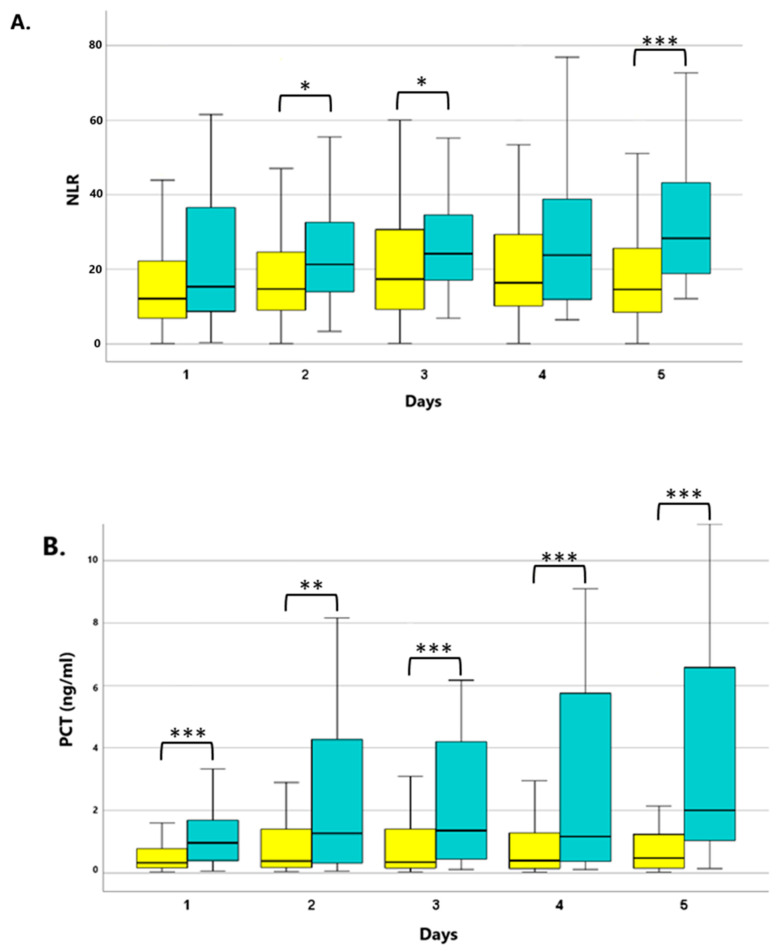
The kinetics of the NLR (**A**) and PCT (**B**) levels between patients with or without the need for renal replacement therapy. Yellow columns: patients without the need for RRT; blue columns: patients with the need for RRT. * *p* < 0.05, ** *p* < 0.01 and *** *p* < 0.005 between the “non-RRT” and “RRT” groups. Data are presented as medians in 25–75% interquartile ranges and 5–95% confidence intervals.

Higher levels of all parameters were measured in patients who required some form of renal replacement therapy. We classified the patients into the “necessity for RRT” group if they required intermittent haemodialysis or continuous renal replacement therapy within the five-day study period. In terms of the development of kidney failure, significantly elevated CRP values were observed on Days 2 and 3 (*p* < 0.05), and consistently significantly higher PCT values (Figure 2A) were measured from Days 1 to 5 (*p* < 0.01; *p* < 0.005). Concerning IL-6, significantly elevated values were found on Days 1, 4, and 5 (*p* < 0.05). Regarding the non-conventional markers, significantly higher NLR values (Figure 2B) were recorded on Day 3 (*p* < 0.05) and Day 5 (*p* < 0.001), whereas no significant differences were found in the PLR between the two groups.

#### 3.1.3. Multivariate Regression Analysis

We conducted a multivariate logistic regression analysis of the study variables (NLR, CRP, PCT, IL-6, and PLR) for each day of the study period (Days 1, 2, 3, 4, and 5) to determine the most significant predictors of mortality (Table 2). On Day 1, procalcitonin (PCT) was a significant predictor, with an odds ratio (OR) of 3.267 (95% CI: 1.014–10.528, *p* = 0.047), indicating a substantial association with mortality. On Day 2, the neutrophil-to-lymphocyte ratio (NLR) was also significantly associated with mortality (OR: 1.058, 95% CI: 1.009–1.110, *p* = 0.021). On Day 3, none of the variables reached statistical significance, as indicated by *p*-values greater than 0.05. On Day 4, both C-reactive protein (CRP) and interleukin-6 (IL-6) emerged as significant predictors, with CRP showing an OR of 1.013 (95% CI: 1.001–1.026, *p* = 0.040) and IL-6 an OR of 1.013 (95% CI: 1.001–1.025, *p* = 0.041). On Day 5, NLR and IL-6 remained significant, with the NLR exhibiting the highest OR across the days (OR: 1.140, 95% CI: 1.036–1.255, *p* = 0.007) and IL-6 showing a consistent association (OR: 1.008, 95% CI: 1.001–1.014, *p* = 0.025). Over the five days analysed, the NLR consistently emerged as a risk factor for mortality, although it was not statistically significant on every day. Specifically, on Days 2 and 5, the NLR showed a significant increase in mortality risk. These findings underscore the evolving role of these biomarkers over time in predicting mortality risk.

#### 3.1.4. ROC Analysis

**Figure 3:**
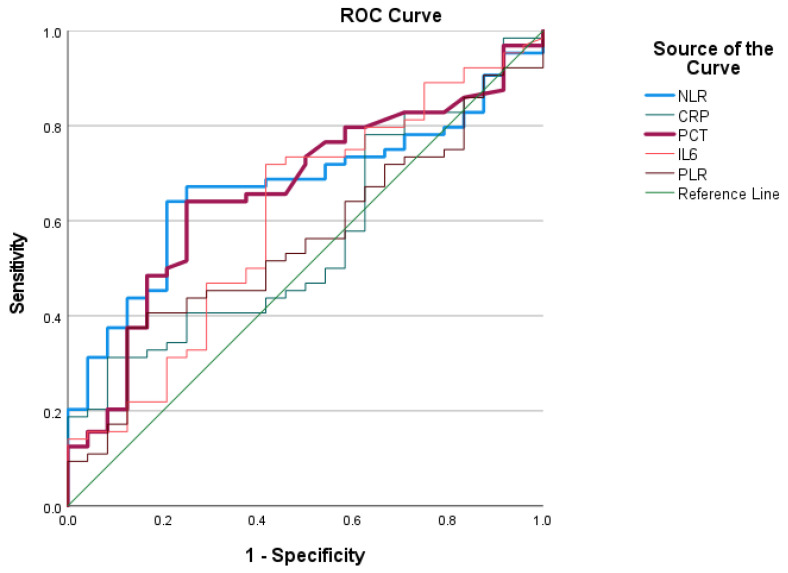
The ROC curve for predicting mortality. The NLR and PCT (highlighted lines) exhibit higher discriminatory power than CRP, IL-6, and the PLR. The reference line (green line) represents a model with no discriminative ability.

We subjected the study parameters to ROC curve analyses to compare their predictive values. In the ROC analyses, we utilised the values measured on the first day to generate statistics and assess the predictive value of all the examined markers in relation to mortality and the need for RRT. The ROC analyses of the four examined markers demonstrated varying levels of discriminatory power in predicting mortality (Figure 3). The NLR had the highest AUC (0.671), with a significant *p*-value (*p* = 0.014), indicating a fair discriminative ability and supporting its role as a useful marker. PCT also showed a notable AUC of 0.662 (*p* = 0.020), further indicating its potential utility in predicting mortality risk. The confidence intervals for the NLR and PCT further support their reliability as predictive markers, as they do not encompass the null value of 0.5, unlike the other markers examined.

We performed another ROC analysis to assesses the predictive value of the same markers for the development of the need for renal replacement therapy. The NLR showed an AUC of 0.615, suggesting moderate predictive power, although it did not reach statistical significance (*p* = 0.127). These findings suggest that within this cohort, none of the tested markers provided strong predictive power for the need for renal replacement therapy, as evidenced by their moderate AUC values and the lack of statistical significance. Further research is necessary to identify more robust biomarkers for predicting the need for renal replacement therapy in COVID-19 patients.

Additionally, we determined the non-conventional markers’ cut-off values based on the ROC curve. For the NLR, the cut-off values were 6.56 (sensitivity: 0.813; specificity: 0.833) for mortality and 6.39 (sensitivity: 0.895; specificity: 0.812) for acute renal failure.

## 4. Discussion

Our objective was to compare the kinetics of three conventional markers routinely used in everyday practice (CRP, PCT, and IL-6) and two non-conventional markers (NLR and PLR) in critically ill COVID-19 patients requiring life-maintaining therapy. The novelty of our study lies in the detailed kinetic analysis of these markers, which provides in-depth insights into their roles in the progression of severe COVID-19. Additionally, the neutrophil/lymphocyte ratio (NLR) has emerged as a significant prognostic marker, with studies showing that higher NLR values are strongly associated with increased disease severity and mortality, providing a reliable tool for early risk stratification in critically ill COVID-19 patients [20]. By integrating these findings with the results of our kinetic analysis, we enhanced the understanding of how these biomarkers can be utilised to better predict and manage severe outcomes in COVID-19. We assume that the increase in IL-6 observed on Days 4 and 5 in the non-survivor patient group may indicate a bacterial superinfection [21].

Our findings indicate significantly elevated NLR values among patients with serious, ventilation-dependent COVID-19 pneumonia, as has been previously reported [22]. The NLR appears to be a reliable predictor of mortality in critically ill patients with confirmed SARS-CoV-2 infection, as PCR testing indicates, and is comparable to the conventionally used PCT and CRP. Based on our findings, and similar to the results obtained by Damar Cakirca et al., we also identified significantly elevated NLR values among patients who developed ARDS via SARS-CoV-2 infection [22]. The significantly increasing trend observed in the deceased group, in contrast to the significantly decreasing values in the survivors, clearly highlights this difference. G. Ponti et al. reported similar results in their publication from May 2020 [23].

Among the COVID-19-infected individuals included in our study, we observed significantly elevated PCT and NLR values in the deceased patient group compared with the survivor group. According to our hypothesis, this could indicate a bacterial superinfection, which, based on previous studies, is associated with a several-fold increase in mortality [24]. In the patients who developed renal failure and required renal replacement therapy during the 5-day study period, we measured significantly higher PCT values (Days 1 to 5) and NLR values (Days 2, 3, and 5). Based on the predictive value of these non-conventionally used markers, such as the NLR, it may be possible to initiate renal replacement therapy earlier and reduce mortality rates. In our study, we consistently identified the NLR as a significant predictor of mortality on several days, particularly on Days 2 and 5, when it demonstrated a protective effect against mortality risk in COVID-19 patients. This aligns with the results from a systematic review and meta-analysis, which reported a high diagnostic value of the NLR for predicting both disease severity and mortality in COVID-19 patients, with an AUC of 0.90 for mortality prediction [20].

It is important to compare the cost of determining the examined markers in everyday practice. At our clinic, a PCT test costs approximately EUR 16, whereas a CRP test costs EUR 2.5 and a complete blood count costs EUR 0.75. In contrast, the determination of the NLR and PLR is based on routine blood tests, eliminating the need for additional expensive laboratory tests or human resources, making their use widely accessible.

## 5. Study Limitations

The main limitation of our study is its single-centre design. Another limitation is the absence of vital patient parameters, such as oxygen saturation, blood pressure, detailed blood gas data, and ventilation parameters, which were not recorded. To ensure that the non-conventional markers that we examined become reliable and routinely used markers, larger sample sizes and multicentre studies are necessary. Additionally, some patients in the survival group were discharged from the ICU, and we did not track their subsequent clinical outcomes. As a result, these patients may have succumbed to long-term complications in other hospital wards. Another limitation of this study is the low proportion and number of patients requiring renal replacement therapy, which prevents us from drawing definitive conclusions from the data. We did not categorize patients with acute kidney failure according to the KDIGO classification. Instead, we grouped the patients based solely on whether renal replacement therapy was required during the study period. To thoroughly predict the need for renal replacement therapy, a multicentre study would be required.

## 6. Conclusions

The generated ratios based on cellular-level inflammatory markers demonstrated predictive value for survival among ICU-dependent COVID-19 patients that is comparable to that of inflammatory markers commonly utilised in current clinical practice. In this study, we analysed the kinetics of non-conventional, “old school”-based “novel” markers, such as the NLR, highlighting their potential to enhance mortality prediction related to viral complications. The advantages of these markers lie in the simplicity of their determination, which does not require highly sophisticated equipment or methodologies, underscoring their cost-effectiveness and applicability in low-income countries. They also allow for the selection of patients who stand to gain the most from complex serodiagnostics, thereby optimising laboratory resources.

## 7. Future Utilisation

The principal conclusion from our study is that the NLR and PCT are the most reliable markers for predicting mortality among critically ill COVID-19 patients admitted to the ICU. The NLR emerged as a consistently significant predictor across all days in our study, with its highest predictive value observed on Day 5. Similarly, PCT demonstrated fair discriminatory power, as indicated by the results of the ROC analyses.

PCT and the NLR were more reliable markers for predicting the need for renal replacement therapies than CRP and the PLR, which were less effective.

The findings suggest that incorporating the NLR and PCT into routine clinical practice could enhance the early identification of high-risk patients, thereby improving patient management and outcomes. Future research should focus on larger, multicentre studies to validate these findings and explore the utility of these markers in various clinical settings. Furthermore, the development of standardised protocols for using these biomarkers in clinical practice could significantly impact the management of COVID-19 and other critical illnesses.

## Figures and Tables

**Table 1:** Comparative analysis of the clinical characteristics of survivors and non-survivors in the patient cohort (N = 167). The parameters analysed included age (median and IQR 95%), gender distribution, obesity, hypertension, diabetes mellitus, chronic obstructive pulmonary disease (COPD), and the utilisation of renal replacement therapy (RRT).

	Survivors N = 39 (%)	Non-Survivors N = 128 (%)	All Patients N = 167 (%)	*p*-Value
Age	65 [30–80]	71 [47.35–85.55]	69 [46–85]	0.667
Male	29 (74)	75 (59)	104 (62)	0.112
Female	10 (26)	53 (41)	63 (38)	0.017
BMI > 30	21 (54)	53 (41)	74 (44)	0.236
Hypertension	34 (87)	57 (45)	91(54)	<0.001
Diabetes mellitus	15 (38)	31 (24)	46 (28)	0.124
COPD	2 (5)	19 (15)	21 (13)	0.185
RRT	0	29 (23)	29 (17)	<0.001

**Table 2:** Logistic regression results: significant predictors of mortality (NLR, CRP, PCT, and IL-6) on Days 1 to 5, including the *p*-value (Sig. (*p*)), odds ratio (OR), and 95% confidence interval for the odds ratio (95% CI for OR).

Day	Marker	Sig. (*p*)	OR	95% CI for OR
1	NLR	0.152	1.037	0.987–1.089
	PCT	0.047	3.267	1.014–10.528
2	NLR	0.021	1.058	1.009–1.110
3	NLR	0.073	1.040	0.996–1.085
	CRP	0.143	1.013	0.999–1.007
4	CRP	0.040	1.013	1.001–1.026
	IL-6	0.041	1.013	1.001–1.025
5	NLR	0.007	1.140	1.036–1.255
	IL-6	0.025	1.008	1.001–1.014

## Data Availability

The data presented in this study are available on request from the corresponding author. The table contains confidential patient data that can only be disclosed to a third party with written consent provided by the patients or their relatives.
